# Clinical Characteristics and Disease Severity Among Infants With SARS-CoV-2 Infection in Montreal, Quebec, Canada

**DOI:** 10.1001/jamanetworkopen.2020.30470

**Published:** 2020-12-14

**Authors:** Luc Panetta, Catherine Proulx, Oliver Drouin, Julie Autmizguine, Thuy M. Luu, Caroline Quach, Fatima Kakkar

**Affiliations:** 1 Division of Infectious Diseases, Department of Pediatrics, Centre Hospitalier Universitaire Sainte-Justine, Montreal, Quebec, Canada; 2 Division of General Pediatrics, Department of Pediatrics, Centre Hospitalier Universitaire Sainte-Justine, Montreal, Quebec, Canada; 3 Department of Pharmacology, Centre Hospitalier Universitaire Sainte-Justine, Montreal, Quebec, Canada

## Abstract

This case series describes clinical characteristics and disease severity in infants who had SARS-CoV-2 infection in Montreal, Quebec, Canada.

## Introduction

With more than 40 million cases of confirmed severe acute respiratory syndrome coronavirus 2 (SARS-CoV-2) infection worldwide, the clinical presentation and risk factors for disease severity among adults and children have now been well described.^1^ SARS-CoV-2 is the virus that causes coronavirus disease 2019. However, little remains known about disease manifestation and severity of illness in infants.^2^ Although infants are at higher risk of severe disease and complications from other common viral respiratory pathogens (influenza, respiratory syncytial virus), it is not yet clear whether this is the case with SARS-CoV-2 infection because few series describing outcomes in infants have been published.^3,4^ The objective of this study was to describe the manifestations and severity of disease among infants with SARS-CoV-2 infection.

## Methods

This case series describes all infants younger than 1 year with laboratory-confirmed SARS-CoV-2 infection (confirmed a by positive reverse transcriptase–polymerase chain reaction result) who were diagnosed or treated at Centre Hospitalier Universitaire Sainte-Justine (CHU-SJ), Montreal, Quebec, Canada, between February 14 and May 31, 2020. The study was approved by the CHU-SJ research ethics board with the requirement for informed consent waived owing to the retrospective nature of the study. Clinical features and severity of disease were compared between infants younger than 3 months chronologic age (younger infants) and infants 3 to 12 months old (older infants). Disease severity was defined using the widely used criteria from Dong et al.^5^ The Appropriate Use and Reporting of Uncontrolled Case Series in the Medical Literature guideline (https://www.ajo.com/article/S0002-9394(10)00690-2/fulltext) was the reporting guideline for this case series. Variables were compared using the Wilcoxon rank sum or Fisher exact test where appropriate, using a 2-sided α level, with *P* < .05 considered statistically significant. Statistical analyses were performed with Stata, version 12.1 software (StataCorp).

## Results

Of 1165 infants who were tested for SARS-CoV-2 infection at CHU-SJ during this 2020 study period, 25 (2%) had confirmed positive results, and 8 of those with positive results (32%) required hospitalization. Two additional infants with SARS-CoV-2 positive results were transferred directly to CHU-SJ for hospitalization and are included in the analysis. Of those 27 infants, 15 (56%) were male, the median age was 89 days (interquartile range [IQR], 34-193 days), and 7 (26%) had comorbid conditions.

Table 1 describes clinical manifestations and disease severity among all infants. Disease was mild in most cases (24 infants [89%]) of cases. The most common presenting symptoms were gastrointestinal tract symptoms (23 infants [85%]), followed by fever (temperature ≥38.0 °C (22 [81%]), and upper respiratory tract symptoms (16 [59%]). There were no significant differences in clinical manifestation between older vs younger infants. However, there was a higher incidence of comorbid conditions among older vs younger infants (6 older infants [46%] vs 1 younger infant [7%]; *P* = .03), including lower birth weight (median [IQR] birth weight, 2055 g [988-2886 g] among older infants vs 3138 g [2998-3505 g] among younger infants; *P* = .02) and gestational age (median [IQR] age at birth, 34.0 wk [30.0-37.0 wk] among older infants vs 38.8 wk [38.5-39.6 wk] among younger infants; *P* = .05). Five infants (19%) had concurrent urinary tract infections caused by *Escherichia coli*.

**Table 1.** Characteristics of 27 Infants With SARS-CoV-2 Infection^a^

Characteristic	No. (%)			*P* value^b^
	Total (N = 27)	Age		
		<3 mo (n = 14)	3-12 mo (n = 13)	
Epidemiologic features				
Chronologic age, median (IQR), d	89 (34-193)	34 (23-65)	233 (148-285)	
Males	15 (56)	7 (50)	8 (62)	.70
Comorbid conditions	7 (26)	1 (7)	6 (46)	.03
Prematurity <37 wk of gestation	6 (22)	1 (7)	5 (38)	.08
Gestational age at birth, median (IQR), wk (n = 15)	38.6 (34.4-39.2)	38.8 (38.5-39.6)	34.0 (30.0-37.0)	.05
Birth weight, median (IQR), g (n = 18)	3073 (2718-3375)	3138 (2998-3505)	2055 (988-2886)	.02
Breastfeeding, No./total No. (%)	10/22 (45)	9/14 (64)	1/8 (13)	<.001
Family contact^c^	14 (52)	8 (57)	6 (46)	.57
Hospitalization	10 (37)	8 (57)	2 (15)	.05
Mild disease^d^	24 (89)	12 (86)	12 (92)	.59
*Escherichia coli* urinary tract infection^e^	5 (19)	4 (29)	1 (8)	.33
Clinical symptoms				
Fever (temperature ≥38 °C)	22 (81)	10 (71)	12 (92)	.32
Hemodynamic signs^f^	12 (44)	9 (64)	3 (23)	.05
Respiratory symptoms				
Upper tract^g^	16 (59)	6 (43)	10 (77)	.12
Lower tract^h^	10 (37)	5 (36)	5 (38)	>.99
Gastrointestinal tract symptoms^i^	23 (85)	13 (93)	10 (77)	.33
Neurologic symptoms^j^	11 (41)	8 (57)	3 (23)	.12
Cutaneous symptoms^k^	4 (15)	2 (14)	2 (15)	>.99
Laboratory and radiologic findings, median (IQR)				
White blood cell count, /μL (n = 18)	8700 (7200-13 600)	10 500 (7600-14 100)	8600 (5500-8600)	.12
Neutrophil count, /μL (n = 18)	2800 (1000-4300)	3100 (2100-8300)	900 (500-1600)	.03
Lymphocyte count, /μL (n = 18)	4700 (3600-6000)	4300 (3200-5800)	5700 (4700-6000)	.32
Platelet count, ×10^3^/μL (n = 18)	303 (260-422)	368 (271-510)	257 (238-276)	.18
ALT, U/L (n = 9)	22 (21-54)	22 (14-54)	72 (22-122)	.46
CRP >10 mg/dL, No./total No. (%)	3/12 (25)	3/9 (33)	0/3	.06
Chest radiograph with mild interstitial anomalies, No./total No. (%)	2/11 (18)	2/7 (29)	0/4	.49

Abbreviations: ALT, alanine aminotransferase; CRP, C-reactive protein; IQR, interquartile range; SARS-CoV-2, severe acute respiratory syndrome coronavirus 2.

SI conversion factors: To convert white blood cell, neutrophil, and lymphocyte counts to ×10^9^/L, multiply by 0.001; platelet count to ×10^9^/L, multiply by 1.0; ALT to μkat/L, multiply by 0.0167; and CRP to mg/L, multiply by 10.

^a^ The characteristics of the analyzed population include those of the 2 patients from other hospitals; however, the hospitalization rate should not be compared with the rate of hospitalization reported in the literature.^6^

^b^ *P* < .05 was considered statistically significant.

^c^ Family contact was defined by a contact with a symptomatic or laboratory-confirmed family member.

^d^ Mild disease was defined using Dong et al criteria.^5^

^e^ Defined by urine culture with greater than 100 000 colony-forming units by catheterization.

^f^ Defined as tachycardia on admission or need for fluid resuscitation.

^g^ Defined as cough, pharyngitis, nasal congestion, rhinorrhea, or sneezing.

^h^ Defined as wheezing, increased work of breathing, tachypnea (respiratory rate, >60/min), apnea, or oxygen saturation less than 94%.

^i^ Defined as abdominal pain, diarrhea, vomiting, increased regurgitation, feeding difficulties, or jaundice.

^j^ Defined as irritability, drowsiness, hypotonia, or seizure.

^k^ Defined as a maculopapular rash on any part of the body.

Clinical characteristics and laboratory and imaging findings of the 10 hospitalized infants are described in Table 2. Three of the hospitalizations (30%) were determined to be non-SARS-CoV-2–related (urinary tract infection, n = 2; intussusception, n = 1). Disease was again mild in most (7 [70%]) of hospitalized patients; none of the infants required supplemental oxygen. The single admission to the intensive care unit was for a non-SARS-CoV-2–related infection (urosepsis from *E coli*).

**Table 2.** Characteristics of 10 Infants Hospitalized With SARS-CoV-2 Infection

Characteristics	No. (%)
Age, median (IQR), d	33 (23-66)
Males	5 (50)
Comorbid conditions	2 (20)
Gestational age at birth, median (IQR), wk	38.5 (33.0-39.1)
Birth weight, median (IQR), g	2950 (1983-3119)
Disease severity	
Mild disease	7 (70)
Length of hospital stay, median (IQR), d	2 (1.0-3.8)
Oxygen therapy	0 (0)
Admission to pediatric intensive care unit	1 (10)
Reason for hospitalization	
Fever without a source in newborn	3 (30)
*Eschericia coli* urinary tract infection^a^	2 (20)
Bronchiolitis	1 (10)
Parental anxiety	1 (10)
Intussusception	1 (10)
Fever (temperature ≥38 °C)	6 (60)
Investigations	
Chest radiography	6 (60)
Normal	4 (67)
Lumbar puncture	5 (10)
Normal	5 (100)
Laboratory parameters^b^	10 (100)
Normal white blood cell count	6 (60)
Normal neutrophil count	3 (30)
Normal lymphocyte count	9 (90)
Normal platelet count	8 (80)
Normal ALT (n = 8)	5 (63)
CRP <10 mg/L	7 (70)

Abbreviations: ALT, alanine aminotransferase; CRP, C-reactive protein; IQR, interquartile range; SARS-CoV-2, severe acute respiratory syndrome coronavirus 2.

^a^ Defined by urine culture with >100 000 colony-forming units by catheterization.

^b^ We used age-adjusted standardized values according to local laboratory reference ranges.

## Discussion

In this case series report, we describe the first series, to our knowledge, of infants with SARS-CoV-2 infection reported from Canada. Our findings are consistent with previous series describing infants who present with mainly fever, mild disease, and no need for mechanical ventilation or intensive care treatment.^3,4^ However, the proportion of hospitalization among the 25 infants tested at our center (32%) was lower than that reported by the US Centers for Disease Control and Prevention (62%) among 95 infants with known hospitalization status.^6^ We suspect this higher risk may reflect practice in the early phase of the pandemic, when in the absence of data, infants may have been hospitalized out of an abundance of caution. These results suggest that no additional SARS-CoV-2–related investigations may be necessary for the majority of infants.

Our study is limited by its small sample size, retrospective observational study design, and testing indications that followed public health guidelines. As a result, we have included only symptomatic infants in our analysis and potentially overestimate the severity of illness.

Clinical signs and disease severity among infants in our series differ from those reported in children and older adults. Our patients had a predominance of gastrointestinal tract symptoms, even in the absence of fever, and mild disease overall. Given these differences, further work should be done to understand the pathophysiological mechanisms underlying the immune response to infection in infants, as this may be a key to addressing the underlying the morbidity associated with SARS-CoV-2 infection in adults.
