# Error in Byline

**DOI:** 10.1001/jamanetworkopen.2021.0356

**Published:** 2021-02-05

In the Research Letter titled “Clinical Characteristics and Disease Severity Among Infants With SARS-CoV-2 Infection in Montreal, Quebec, Canada,”^1^ published December 14, 2020, an error occurred in the byline such that the third author’s first name was misspelled. His full name should have appeared as “Olivier Drouin, MDCM, MSc, MPH.” This article has been corrected.^1^
